# Cystoid macular edema prophylaxis in cataract surgery: A protocol for network meta-analysis

**DOI:** 10.1371/journal.pone.0314467

**Published:** 2024-12-17

**Authors:** Keean Nanji, Phillip Staibano, Tyler McKechnie, Michael Zoratti, Varun Chaudhary

**Affiliations:** 1 Department of Surgery, Division of Ophthalmology, McMaster University, Hamilton, Ontario, Canada; 2 Department of Health Research Methods, Evidence and Impact, McMaster University, Hamilton, Ontario, Canada; 3 Department of Surgery, Division of Otolaryngology–Head and Neck Surgery, McMaster University, Hamilton, Ontario, Canada; 4 Department of Surgery, Division of General Surgery, McMaster University, Hamilton, Ontario, Canada; Aravind Eye Hospital, INDIA

## Abstract

**Objective:**

Cataracts are the leading cause of global preventable and treatable blindness. Cystoid macular edema (CME) is among the most common complications following cataract surgery. The development of CME impacts patients’ quality of life and has economic implications for patients and healthcare systems. The purpose of this review is to synthesize the evidence from randomized controlled trials evaluating patients receiving prophylactic treatment with nonsteroidal anti-inflammatory drugs or corticosteroid medications to determine the comparative effectiveness of each specific regimen on retinal thickness, visual acuity, the development of CME, patient quality of life, intraocular pressure and adverse events following uncomplicated, age-related cataract surgery performed by phacoemulsification.

**Methods:**

A systematic review and random effects Bayesian network meta-analysis (NMA) will be performed and will be reported following the Preferred Reporting Items for Systematic Reviews and Meta-Analysis (PRISMA) extension statement for NMA. A comprehensive electronic search will be performed of the MEDLINE, EMBASE and CENTRAL databases, as well as of the ClinicalTrials.gov and World Health Organization International Clinical Trials Registries. Data will be collected and synthesized for seven pre-specified outcomes at 6 weeks and 3 months following surgery: i) change in central retinal thickness measured by optical coherence tomography (OCT), ii) best-recorded visual acuity iii) the rate of CME measured by OCT, and fluorescein angiography (FA) iv) the rate of patients experiencing clinically significant macular edema defined as the presence of CME and pre-specified thresholds for decreased visual acuity, v) patient quality of life, vi) intraocular pressure and vii) the number of patients experiencing one or more pre-specified adverse events. The certainty of evidence for each outcome will be assessed using GRADE NMA guidelines.

**Discussion:**

The results of this NMA will provide a comprehensive evaluation of the evidence for this critical question with significant clinical equipoise.

**Trial registration:**

**Systematic Review Registration:** PROSPERO Registration Number: CRD42024531150.

## Introduction

### Background and rationale

Cataracts are the leading cause of global preventable and treatable blindness [1]. Cataract surgery is one of the most frequently performed surgical procedure globally with an estimated 20 million cases performed per year [2,3]. Cystoid macular edema (CME) is among the most common causes of visual impairment following cataract surgery with prevalence rates varying between 0.9–23% depending on the diagnosis method used and on the presence of patient risk factors [4,5]. The pathophysiology of CME is not fully understood but has been hypothesized to occur due to post-operative inflammation facilitating disruption of the blood-retinal barrier and leakage from the perifoveal retinal capillaries [6,7]. Patients who develop CME may experience, decreased vision, reduced contrast sensitivity, metamorphopsia and/or central scotomas [7]. While CME can be treated, patients may experience permanent vision loss after the resolution of edema due to changes in the photoreceptor architecture [8,9]. Moreover, the development of CME increases the costs associated with cataract surgery by an estimated 47% [10]. Given the frequency of cataract surgery, even small improvements in perioperative care can have substantial clinical and economic effects.

Nonsteroidal anti-inflammatory drugs (NSAIDs) and corticosteroid drugs are frequently used to prevent CME [5,11]. A 2016 Cochrane review concluded that topical NSAIDs may reduce the risk of CME but that it was unclear if there was any impact on visual function or quality of life; the value of adding NSAIDs to steroids was also deemed uncertain [12]. Since this review, multiple large randomized controlled trials (RCTs) have attempted to address the evidence gap [13–15]. Additionally, since this review, multiple RCTs have evaluated the safety and efficacy of subtenon steroids as an alternative to topical therapy [13,16]. Previous reviews evaluating prophylactic interventions for the development of CME have been narrative or focused solely on direct comparisons limiting the clinical applications that can be generated [5,12,17]. The lack of high-quality evidence is reflected by the considerable variability amongst surgeons and institutions regarding prophylactic practice patterns [7].

The importance of an evidence-based protocol will only be magnified given the projected increase in the global burden of cataracts over the next 15 years [18]. There is a critical need for an updated systematic review and meta-analysis to evaluate the impact of available prophylactic treatment options for the development of CME following cataract surgery. Given that there are multiple potentially efficacious agents, a network meta-analysis (NMA) is the ideal statistical method for investigating this topic [19–22]. Unlike a traditional pairwise meta-analysis, NMA facilitates comparisons across three or more interventions. NMA enables the inclusion of additional published trials by integrating both direct and indirect evidence into one model to provide more precise effect estimates and the relative effectiveness of competing interventions [21–23].

### Objectives

The focus of this investigation will be to address the following question: **In patients over the age of 18 randomized to receive prophylactic treatment to prevent CME following uncomplicated, age-related cataract surgery performed by phacoemulsification, what is the comparative effectiveness of each specific regimen of NSAIDs and/or corticosteroid therapy on the incidence of CME, retinal thickness, visual acuity, patient quality of life, intraocular pressure and adverse events at 6 weeks and 3 months following surgery?** This investigation will evaluate all ocular routes and timing of NSAID and corticosteroid treatment (topical NSAIDs, topical corticosteroids, subtenon steroids, subconjunctival steroids, intravitreal steroids, and intravitreal steroid implants).

## Methods

The systematic review and NMA will be reported according to the Preferred Reporting Items for Systematic Reviews and Meta-Analysis (PRISMA) extension statement for NMA [17]. The protocol has been reported following the PRISMA-Protocol extension statement (S1 Table) [24,25], and has been registered on PROSPERO (CRD42024531150). This study is exempt from ethics approval as all syntheses will be performed utilizing previous clinical trial data for which informed consent and ethics approval have been obtained. S1 File displays the estimated timeline for each phase of the review process.

### Eligibility criteria

We utilized the PICOTS framework to design the framework for the eligibility criteria [26]. Table 1 outlines these criteria:

**Table 1:** Eligibility criteria.

Domain	Inclusion Criteria	Exclusion Criteria
Population	• Patients with an age-related cataract randomized to receive treatment to prevent CME following uncomplicated, cataract surgery performed by phacoemulsification.• In keeping with a Cochrane review from 2016, we will include participants irrespective of their baseline risk of CME; particularly of note, we will be including individuals with diabetes and uveitis. [27–29]	• The focus of this review is to evaluate patients undergoing phacoemulsification. We will exclude patients undergoing cataract surgery performed using extracapsular cataract extraction, manual small incision cataract surgery, or intracapsular cataract extraction [30,31]. Thus, per methodologic guidelines, if a study includes a population in which greater than 20% of patients underwent cataract surgery in a method other than phacoemulsification and the study does not present data for the subgroup of patients without diabetes or uveitis, the study will be excluded [32].
Interventions	• The interventions of interest will be patients receiving either topical NSAIDs, topical corticosteroids, subtenon steroids, subconjunctival steroids, intravitreal steroids, intravitreal steroid implants, or a combination of these treatments either pre-operatively and post-operatively or solely post-operatively for the prevention of CME. • To facilitate analysis, as has been done with previous reviews on the topic [12,17], we will group individual medications according to their class and route.	The focus of this review is to evaluate the ability of interventions as prophylaxis agents, not to treat CME. We will be excluding any interventions that are used to treat patients with CME.
Comparators	• The comparator group of interest will be placebo. • To be eligible, studies may compare any of the interventions of interest to either each other or to a placebo.	
Outcomes	• This review will evaluate seven pre-specified outcomes that are important to patients, clinicians, and decision-makers: 1) The change in a measure of the central retinal thickness as measured by optical coherence tomography (OCT) compared to the pre-operative measurement. 2) Best-recorded visual acuity (BRVA) following cataract surgery. 3) The rate of CME following surgery measured a) by OCT, or b) by fluorescein angiography (FA). 4) The rate of patients experiencing clinically significant macular edema as defined by the presence of CME on OCT or FA along with a documented pre-specified decrease in vision [14,33]. 5) Patient quality of life assessed using a validated measure. The three most frequently used patient reported outcome measures in cataract surgery research are: The National Eye Institute Vision Function Questionnaire 25 (NEI-VFQ-25), the EuroQol EQ-5D, and the Catquest-9SF [34]. As such, in the presence of sufficient data, we will meta-analyse the results from each of these measures individually. In the absence of sufficient data for this analysis, results will be combined and meta-analyzed utilizing standardized mean differences. 6) Patients’ intraocular pressure. 7) The number of patients experiencing one or more of the following adverse event(s): IOP increase ≥ 10 mmHg from baseline, IOP readings ≥ 25 mmHg, or vision loss ≥ 15 letters compared to baseline.	
Time	• The incidence of CME typically occurs within 3 months of surgery with a peak incidence 4–6 weeks post-operatively [35]. • Thus, we will assess each of the seven outcomes at 6 weeks, and at 3 months. • The six-week outcomes will include any outcome reported between 4 and 8 weeks; the 3-month outcomes will include any outcome reported between 8 weeks and 16 weeks.	• Given that the focus of this review is on the incidence of CME rather than the treatment of CME, we will exclude studies only reporting outcomes after 16 weeks.
Study Design	• This review will only include RCTs.	

### Information sources

Searches of Ovid MEDLINE, Ovid EMBASE, and CENTRAL databases as well as the ClinicalTrials.gov and World Health Organization International Clinical Trials registries were performed from inception until February 27^th^ 2024, for RCTs meeting the eligibility criteria. The sensitive search strategy was devised collaboratively with a multidisciplinary research team consisting of an academic research librarian and clinicians. To identify any potentially missing studies, the electronic search will be supplemented by hand-searching the reference lists from included studies, and by contacting clinical experts in the field. Captured citations will be exported to Covidence (Veritas Health Innovation, Melbourne, Australia) for further screening.

### Search strategy

The search strategy for each database and registry is displayed in S2 File.

### Selection process

Two reviewers working independently and in duplicate, will review the titles and abstracts of search records and subsequently all full texts to determine eligibility. All disagreements will be resolved by discussion and, when not possible, through adjudication by a third party. Covidence (Melbourne, Australia) will be utilized to track triage decisions.

### Data extraction

Following training and calibration, pairs of reviewers will work independently and in duplicate to complete the extraction. The calibration process is outlined in S1 File. The following information was extracted: the study design, the date the study was conducted, the number of participants in each group, the mean age of participants, the sex of participants, baseline characteristics of the study populations including the percentage of patients with diabetes and uveitis, the study inclusion criteria, details regarding the intervention agents used, the primary and secondary outcomes of each trial including methods in which the outcomes were assessed, the follow-up period of each trial, and whether the trial received funding. Any unclear information from the published manuscripts will be clarified with the corresponding author.

### Risk of bias

Following training and calibration, pairs of reviewers, working independently and in duplicate, will assess the risk of bias for each eligible trial for each outcome of interest utilizing the Cochrane Risk of Bias Version 2 tool [36]. Disagreements will be resolved through discussion and, when not possible, through adjudication by a third party. For each eligible outcome, the risk of bias will be rated as ‘low,’ ‘some concerns’, or ‘high’ based on assessments across the following domains: the randomization process, deviations from the intended interventions, missing outcome data, measurement of the outcome, and selection of the reported result.

### Statistical analysis

We will present the results of dichotomous outcomes as odds ratios (ORs) and continuous outcomes as mean differences (MDs) or standardized mean differences (SMDs), with the associated 95% credible interval (CrI). For each comparison, we will perform a Bayesian network meta-analysis with random effects modelling. Estimates will be obtained using the Markov Chains Monte Carlo (MCMC) method with non-informative priors. 5,000 initial iterations will be used for adaptation, followed by 100,000 iterations for the calculation of odds ratios or MDs and their corresponding 95% CrI. Convergence will be assessed via the Brooks-Gelman-Rubin statistic [37].

Complete case analyses will be performed for all analyses. In the presence of sufficient data, analyses will be performed in three separate manners with progressively stricter criteria for defining each group. The first analysis will group patients by drug class and route of administration irrespective of the timing and dosing of the medication (e.g. patients receiving topical NSAIDs given pre-operatively and post-operatively would be combined with patients receiving topical NSAIDs solely post-operatively). The second analysis will group patients by drug and route of administration but will separate groups depending on the timing of the drug (e.g. patients receiving topical NSAIDs pre-operatively and post-operatively would be separate from patients receiving topical NSAIDs solely post-operatively). Lastly, an analysis will be performed in which drugs will be separated within each class and grouped solely by modality and peri-operative timing (e.g. topical diclofenac given post-operatively and topical ketorolac given post-operatively would inform separate nodes).

If there is insufficient evidence to perform a network meta-analysis for a particular outcome, a pairwise meta-analysis will be conducted. If there’s insufficient evidence for a pairwise analysis, then results will be synthesized narratively.

We will use node-splitting models to assess local incoherence [38]. Heterogeneity between RCTs for each direct comparison will be assessed with visual inspection of forest plots and the I^2^ statistic. Heterogeneity of 0% to 40% will be considered as “might not be important,” 30% to 60% as “moderate heterogeneity,” 50% to 90% as “substantial heterogeneity,” and 75% to 100% as “considerable heterogeneity”.

In the event of missing measures of variability for continuous outcome measures, standard deviations will be imputed in line with the methodology from the Cochrane Handbook of Systematic Reviews of Interventions [39].

All analyses will be performed in R 4.0.3 (The R Project, Auckland, New Zealand) using the “gemtc”, “rjags”, and “dmetar”, and “meta” packages [40–43].

### Subgroup analyses

We will conduct three pre-specified subgroup analyses. The groups of interest will be i) comparing patients with known diabetes at the time of randomization to those without diabetes, ii) comparing patients with a history of uveitis prior to cataract surgery to those without uveitis and iii) comparing individuals who receive femtosecond laser-assisted cataract surgery (FLACS) to those not undergoing FLACS. We hypothesize that there will be a higher rate of CME among patients with diabetes and patients with uveitis and that there will be no difference between patients undergoing FLACS [27–29]. Moreover, we hypothesize that NSAIDS either alone or in combination will be superior to steroids in patients with diabetes in preventing CME, retinal thickening and in improving visual acuity [11,44]. The method of subgroup analysis will depend on the availability of data. In the event of sufficient data, a network meta-regression will be performed evaluating the percentage of patients from each trial with the characteristic of interest. If there are insufficient data for the network meta-regression, the subgroup analysis will compare the pooled effect estimates between studies with and without the characteristic of interest, The Instrument to assess the Credibility of Effect Modification Analyses (ICEMAN tool) will be used to evaluate the credibility of subgroup analysis [45].

### Confidence in cumulative evidence

We will utilize a minimally contextualized approach to assess the effect of the different interventions on the pre-specified outcomes [46]. The certainty of the evidence will be assessed following Grade of Recommendations, Assessment, Development and Evaluation (GRADE) guidelines [47,48]. The GRADE approach involves separate grading of the quality of evidence for each outcome followed by determining an overall quality of evidence across outcomes. Two reviewers will rate each domain for each comparison separately and resolve discrepancies by consensus. The certainty for each comparison and outcome will be rated as ‘high’, ‘moderate’, ‘low’ or ‘very low’, based on considerations of risk of bias, inconsistency, indirectness, publication bias, intransitivity, incoherence, and imprecision. Publication bias will be assessed using comparison-adjusted funnel plots and Egger’s test. For each outcome, we will present the most credible estimate from the NMA or direct pairwise comparison.

### Sensitivity analysis

A sensitivity analysis will be performed excluding studies rated to have a high overall risk of bias. Additionally, a sensitivity analysis will be performed excluding studies for which any data was imputed to conduct the meta-analysis [39].

### Treatment rankings

A strength of NMA is that the analysis can facilitate ranking the different treatments across outcomes. Traditional ranking systems such as the Surface Under the Cumulative Ranking curve (SUCRA) or P-value rankings do not account for the certainty of evidence or the precision of the ranking estimates [49]. Given these limitations, results will be interpreted using partially contextualized methods and presented across outcomes in a ranking incorporating both their relative magnitude of effect and certainty of evidence [46,50].

## Discussion

There is a critical gap in the literature evaluating prophylactic methods for the development of CME following cataract surgery performed by phacoemulsification. This gap is reflected by the significant heterogeneity in surgeons’ practice patterns, and by the lack of clarity in current guidelines; [7,51] the American Academy of Ophthalmology’s most recent preferred practice pattern states that there currently is no firmly established protocol for preventing post-operative CME [51]. An updated assessment of the available certainty of evidence is needed given the relatively recently available large RCTs on the topic, the frequency with which cataract surgery is performed globally, the projected increase in the global burden of cataracts, and the near 50% increase in the costs associated with cataract surgery in patients developing CME [2,3,10,13,14,18,52–54].

NMA is an extension of classical pairwise analyses which combines direct evidence from head-to-head comparisons with indirect evidence obtained through one or more common comparators. This statistical approach facilitates the comparison of treatments that have not been directly compared in head-to-head RCTs, can improve the precision of the effect estimate and can facilitate ranking treatments to better inform clinical decision-making [21,23]. NMA is the preferred statistical approach to answer the research question at hand, given that there are multiple efficacious treatments.

Limitations of the future investigation include potential heterogeneity in study designs, potential differences in outcome definitions across the included studies, and the inherent limitations of NMAs, notably, a reliance on the transitivity assumption. The outlined pre-specified methodology details the steps that will be taken to mitigate the effect of these limitations, such as clear, detailed eligibility criteria and key subgroup and sensitivity analyses.

In conclusion, this protocol outlines the detailed methodology for a systematic review and NMA of RCTs to help answer a patient important question with clinical equipoise. This investigation will provide the most up-to-date effect estimates of the commonly employed prophylactic treatments for the development of CME following phacoemulsification with assessments of the associated certainty of evidence. The results of this investigation have the potential to impact future clinical practice guidelines and clinical decision-making regarding the approach to CME prophylaxis in cataract surgery.

## Supporting information

S1 Table PRISMA-protocol checklist. (DOC)

S1 File Estimated timeline for the review process. (DOCX)

S2 File Search strategy for each database and registry. (DOCX)

## Abbreviations

CME: Cystoid macular edema
RCT: Randomized controlled trial
PRISMA: Preferred Reporting Items for Systematic Reviews and Meta-Analyses
OCT: Optical coherence tomography
FA: Fluorescein angiography
GRADE: Grade of Recommendations, Assessment, Development, and Evaluation
NMA: Network meta-analysis
NSAID: Nonsteroidal anti-inflammatory drug
NEI-VFQ-25: National Eye Institute Vision Function Questionnaire 25
IOP: Intraocular Pressure
OR: odds ratio
MD: mean differences
CrI: Credible interval
MCMC: Markov Chains Monte Carlo
FLACS: femtosecond laser-assisted cataract surgery
ICEMAN: Instrument to assess the Credibility of Effect Modification Analyses
SUCRA: Surface Under the Cumulative Ranking Curve
