# Supplementary material for: Cystoid macular edema prophylaxis in cataract surgery: A protocol for network meta-analysis
Source: PLoS One. 2024 Dec 17;19(12):e0314467. doi: 10.1371/journal.pone.0314467 (PMC11651568; doi:10.1371/journal.pone.0314467)
Supplement: S1 File — (DOCX) [file pone.0314467.s002.docx]

**Supplemental Material 1.**

**Estimated Timeline for the Review Process**

| **Review Stage** | **Estimated Completion Date** |
| --- | --- |
| Drafting of Protocol | February 2024 |
| Search Strategy | February 27^th^ 2024 |
| Title and Abstract Screening | March 31^st^ 2024 |
| Full Text Screening | April 30^th^ 2024 |
| Data Extraction | May 31^st^ 2024 |
| Risk of Bias Assessments | June 30^th^ 2024 |
| Data Analysis | July 31^st^ 2024 |
| GRADE Certainty of Evidence Assessments | August 31^st^ 2024 |
| Writing of Manuscript | November 15^th^ 2024 |
| Submission of Final Manuscript | December 1^st^ 2024 |

**Summary of Training and Calibration Process**

All reviewers involved in the study performed calibrations of each stage of the review process (Title and abstract screening, full-text screening, data extraction, risk of bias assessment and GRADE ratings). All authors had prior training in research methods, and thus, the following calibration process was felt to be sufficient: all authors performed 20 title and abstract screenings, 10 full-text screenings, 5 data extractions, 5 risks of bias assessments and 5 GRADE ratings. The authors subsequently met to discuss any discrepancies and possible areas of confusion. Before completing each stage of the calibration, each author confirmed that they felt comfortable proceeding.
