# Supplementary material for: Cystoid macular edema prophylaxis in cataract surgery: A protocol for network meta-analysis
Source: PLoS One. 2024 Dec 17;19(12):e0314467. doi: 10.1371/journal.pone.0314467 (PMC11651568; doi:10.1371/journal.pone.0314467)
Supplement: S2 File — (DOCX) [file pone.0314467.s003.docx]

**Supplemental Material 2.** Search Strategy for Each Database and Registry

**OVID Medline Epub Ahead of Print, In-Process & Other Non-Indexed Citations, Ovid MEDLINE(R) Daily and Ovid MEDLINE(R) 1946 to Present**

1 Macular Edema, Cystoid.mp. or Macular Edema/

2 CME.mp.

3 CMO.mp.

4 PCME.mp.

5 PCMO.mp.

6 Irvine-gass.mp.

7 1 or 2 or 3 or 4 or 5 or 6

8 Cataract Extraction/ or Cataract/ or Cataract.mp.

9 Lens Implantation, Intraocular/ or Cataract Extraction/ or lens implantation.mp. or Phacoemulsification/ or Lenses, Intraocular/

10 intraocular lens.mp.

11 phaco.mp.

12 Pseudophakia/ or pseudophak*.mp.

13 8 or 9 or 10 or 11 or 12

14 7 and 13

15 randomized controlled trial.pt.

16 randomized.mp.

17 placebo.mp.

18 15 or 16 or 17

19 14 and 18

**Embase**

1 retina macula cystoid edema/

2 cystoid macular edema.mp.

3 macula* edema.mp.

4 macular edema/

5 CME.mp.

6 CMO.mp.

7 PCME.mp.

8 PCMO.mp.

9 Irvine-gass.mp.

10 1 or 2 or 3 or 4 or 5 or 6 or 7 or 8 or 9

11 cataract extraction/ or senile cataract/ or cataract/ or Cataract.mp.

12 intraocular lens.mp. or lens implant/

13 phacoemulsification.mp. or phacoemulsification/ or lens implantation/

14 phaco.mp.

15 Pseudophakia.mp. or pseudophakia/

16 Pseudophakic.mp.

17 11 or 12 or 13 or 14 or 15 or 16

18 10 and 17

19 random:.tw.

20 placebo:.mp.

21 double-blind:.tw.

22 19 or 20 or 21

23 18 and 22

**Database: CENTRAL**

ID Search Hits

#1 MeSH descriptor: [Macular Edema] explode all trees

#2 cystoid macular edema

#3 CME

#4 CMO

#5 Macular edema

#6 macular oedema

#7 PCME

#8 PCMO

#9 #1 OR #2 OR #3 OR #4 OR #5 OR #6 OR #7 OR #8

#10 MeSH descriptor: [Cataract Extraction] explode all trees

#11 MeSH descriptor: [Phacoemulsification] explode all trees

#12 MeSH descriptor: [Lens Implantation, Intraocular] explode all trees

#13 MeSH descriptor: [Pseudophakia] explode all trees

#14 Cataract

#15 Phacoemulsification

#16 Intraocular lens

#17 Pseudophakia

#18 Lens implantation

#19 #10 OR #11 OR #12 OR #13 OR #14 OR #15 OR #16 OR #17 OR #18

#20 #9 AND #19

**Registries:**

**ClinicalTrials.gov:**

Macula Edema, Cystoid OR Macular Oedema, Cystoid OR Irvine Gass OR PCME OR PCMO OR CME OR CMO OR (Macular Edema AND Cataract OR Intraocular lens OR Phacoemulsification OR Pseudophakia)

**WHO ICTRP:**

Macular edema, cystoid OR Cystoid macular edema OR Irvine GASS OR PCME OR PCMO OR CME OR CMO OR (Macular edema and (Cataract or Intraocular lens OR phacoemulsification OR Pseudophakia))
